# The Influence of Nutritional Supplementation for Iron Deficiency Anemia on Pregnancies Associated with SARS-CoV-2 Infection

**DOI:** 10.3390/nu14040836

**Published:** 2022-02-16

**Authors:** Mihaela Uta, Radu Neamtu, Elena Bernad, Adelina Geanina Mocanu, Adrian Gluhovschi, Alin Popescu, George Dahma, Catalin Dumitru, Lavinia Stelea, Cosmin Citu, Felix Bratosin, Marius Craina

**Affiliations:** 1 Discipline of Obstetrics and Gynecology, “Victor Babes” University of Medicine and Pharmacy, 300041 Timisoara, Romania; utamiha@yahoo.com (M.U.); ebernad@yahoo.com (E.B.); adelinaerimescu@yahoo.com (A.G.M.); adigluhovschi@yahoo.com (A.G.); alinp22@yahoo.com (A.P.); george_dahma@yahoo.com (G.D.); dumcatal@yahoo.com (C.D.); stelea_lavinia@yahoo.com (L.S.); citu.ioan@umft.ro (C.C.); crainamariuslucian@gmail.com (M.C.); 2 Methodological and Infectious Diseases Research Center, Department of Infectious Diseases, “Victor Babes” University of Medicine and Pharmacy, 300041 Timisoara, Romania; felix.bratosin7@gmail.com

**Keywords:** SARS-CoV-2, COVID-19, pregnancy, pregnant women, iron deficiency, anemia, nutritional supplementation

## Abstract

Anemia is a very common occurrence during pregnancy, with important variations during each trimester. Anemia was also considered as a risk factor for severity and negative outcomes in patients with SARS-CoV-2 infection. As the COVID-19 pandemic poses a significant threat for pregnant women in terms of infection risk and access to care, we developed a study to determine the impact of nutritional supplementation for iron deficiency anemia in correlation with the status of SARS-CoV-2 infection. In a case-control design, we identified 446 pregnancies that matched our inclusion criteria from the hospital database. The cases and controls were stratified by SARS-CoV-2 infection history to observe the association between exposure and outcomes in both the mother and the newborn. A total of 95 pregnant women were diagnosed with COVID-19, having a significantly higher proportion of iron deficiency anemia. Low birth weight, prematurity, and lower APGAR scores were statistically more often occurring in the COVID-19 group. Birth weight showed a wide variation by nutritional supplementation during pregnancy. A daily combination of iron and folate was the optimal choice to normalize the weight at birth. The complete blood count and laboratory studies for iron deficiency showed significantly decreased levels in association with SARS-CoV-2 exposure. Puerperal infection, emergency c-section, and small for gestational age were strongly associated with anemia in patients with COVID-19. It is imperative to screen for iron and folate deficiency in pregnancies at risk for complications, and it is recommended to supplement the nutritional intake of these two to promote the normal development and growth of the newborn and avoid multiple complications during pregnancy in the COVID-19 pandemic setting.

## 1. Introduction

According to the World Health Organization, more than 2 billion people worldwide suffer from iron deficiency, and more than 38% of pregnant women worldwide suffer from anemia during pregnancy [1]. Anemia affects only 20% of pregnancies in the United States due to intensive screening and empiric nutritional supplementation during pregnancy [2], but still, iron-deficiency anemia is the most frequent anemia of pregnancy and one of the most frequent complications during pregnancy in developed countries [3,4]. Moreover, other studies researching anemia in pregnancy discovered that 42% of randomly chosen non-anemic first trimester women were iron deficient using conventional transferrin saturation and serum ferritin cut-off values [5]; however, iron deficiency screening using serum ferritin testing is not regularly recommended in unselected pregnancies in the United States and the United Kingdom.

Iron deficiency is widespread, especially in women of childbearing age, and is mostly caused by menstrual blood loss and a lack of iron-rich foods consumed orally [6]. This problem is exacerbated during pregnancy. Women need iron and folate throughout pregnancy to satisfy their own demands and those of the growing fetus. The issue is that if pregnant women become lacking in certain nutrients, they will be unable to provide them to the fetus in appropriate amounts. Iron deficiency anemia is defined by a low serum ferritin level, typically 15 µg/L. Additionally, a serum ferritin concentration of 30 µg/L suggests depleted iron reserves [7]. Fetal growth requires around 800–850 mg of iron [8]. Women who are already iron deficient and anemic throughout early pregnancy will exhaust residual iron reserves and become more anemic. In women who are not anemic but are iron deficient, continued depletion of iron reserves may result in anemia. Even women with adequate hemoglobin and iron reserves are at risk of developing an iron deficit later in pregnancy. Iron deficiency anemia is related to an increased risk of blood transfusion, preterm birth, cesarean delivery, and neonatal critical care unit hospitalization if present at delivery [9,10]. Low folate intake before conception raises the chance of neural tube abnormalities in the infant. Inadequate iron and folate levels in women may result in anemia, which makes women weary, dizzy, and more susceptible to infections, such as the SARS-CoV-2, during the ongoing pandemic. As such, iron supplementation is suggested if this problem is detected during pregnancy or after delivery, since it is related to unfavorable maternal and newborn outcomes [11]. Therefore, three possible ways to prevent and control the development of iron deficiency and iron deficiency anemia should be considered. These encompass dietary diversification, food fortification and individual oral iron supplementation, as the first line method [12]. Considering the gastrointestinal upset caused by iron supplementation, this can be administered every other day with similar efficiency [13].

The pandemic caused by SARS-CoV-2 had a dramatic influence on healthcare systems, social institutions, and the global economy [14]. The COVID-19 pandemic’s detrimental impacts on maternal and perinatal health are not confined to the disease’s direct morbidity and death. We expect that Romanian pregnant women who are left behind for prenatal monitoring and treatment as a result of the COVID-19 pandemic’s limitations [15,16,17,18] would have worse pregnancy outcomes, as it was recently demonstrated in a global analysis concluding that maternal and fetal outcomes have deteriorated as a result of the COVID-19 pandemic, with an increase in maternal deaths, stillbirths, and maternal depression [19,20,21,22]. As growing concerns during the pandemic affect medical workers and mothers, we believe that anemia during pregnancy can be easily overlooked in these times, as much as it is still an understudied topic in correlation with COVID-19. Therefore, we aimed to identify potential unwanted outcomes of anemia during pregnancy that might be associated with maternal exposure to SARS-CoV-2 and determine the difference made by nutritional supplementation in these pregnancies.

## 2. Materials and Methods

Between 1 January 2020 and 1 January 2022, the current retrospective population-based cohort research was conducted at a tertiary hospital in Timisoara, Romania, at the Department of Obstetrics and Gynecology affiliated with Timis County Emergency Clinical Hospital. The following criteria applied to pregnant women included in the study: (1) providing informed consent and agreeing to participate; (2) giving birth at our clinic to a live child or stillbirth; (3) having a history of pregnancy screening at our outpatient clinic, with at least one full blood workup; and (4) being tested for SARS-CoV-2 before or during hospitalization using the usual RT-PCR protocol. We eliminated 214 pregnant women with a follow-up history at the outpatient clinic who delivered in a private setting. Additionally, 109 pregnant women who gave birth in our clinic during the study period did not agree to be involved in this research. At the conclusion of the data collection, 351 pregnant women who tested negative for COVID-19 and 95 pregnant women who tested positive for COVID-19 satisfied the inclusion criteria.

Anemia during pregnancy was defined by international guidelines as a hemoglobin concentration (Hb) of 11.0 g/dL in the first trimester, 10.5 g/dL in the second and third trimesters, and 10.0 g/dL postpartum [23]. We also assessed the serum ferritin as an excellent measure of the body’s iron storage, and its level during early pregnancy is typically a solid predictor of iron deficiency, having a reference range of 10 to 200 ng/mL in women [24]. Other variables that were of interest for this particular study comprised general maternal characteristics (age, gravidity, parity), maternal pregnancy complications, neonatal characteristics, a maternal full blood count, sideremia, transferrin, serum iron, total iron-binding capacity (TIBC), a neonatal full blood count, with the additional evaluation of direct and total bilirubin, birth weight, and neonatal complications (prematurity, neonatal death, congenital anomalies, RBC transfusions). Following the diagnosis of iron deficiency anemia and/or folate deficiency, all pregnant patients received a normal release iron supplement with a medium daily dose of iron between 30 mg and less than 60 mg elemental iron. Folic acid was administered in a dose of 400 to 600 micrograms according to the guidelines of the American College of Obstetricians and Gynecologists [25].

This case-control study was approved by the Local Committee of Ethics for Scientific Research of Timis County Emergency Clinical Hospital for the Obstetrics and Gynecology Department, operating under provisions of article 167 of the Law number 95 from 2006, art. 28, chapter VIII of order 904/2006 and with EU GCP Directives 2005/28/EC established at the International Conference on Harmonization of Technical Requirements for Registration of Pharmaceuticals for Human Use (ICH), and with the Declaration of Helsinki—Recommendations Guiding Medical Doctors in Biomedical Research Involving Human Subjects. The current study protocol received ethical approval on 9 December 2021, with approval number A79.

Using the IBM SPSS v.26 statistical software and MedCalc v.20, we analyzed clinical and laboratory data from obstetric patients with and without an associated COVID-19 infection, stratified by the presence of iron deficiency anemia. Normally distributed parametric data were reported as mean and standard deviation, using the student’s t-test or the ANOVA test to test for statistical significance. Non-normal data, as defined by median and interquartile range, were analyzed with the Mann–Whitney U-test and Kruskal-Wallis analysis of variance. The Chi-square test and Fisher’s exact test were used to compare proportions. The significance threshold was set for α = 0.05.

## 3. Results

The general characteristics of study participants presented in Table 1 identified several important differences between groups stratified by COVID-19 status during pregnancy. Anemia was more often present in pregnant mothers with a history of SARS-CoV-2 infection during pregnancy (42.1% vs. 29.3%, *p* = 0.018), and their nutritional supplementation differed significantly. For example, the proportion of pregnant mothers with COVID-19 who were self-medicated or medically prescribed iron and folate as daily supplements were statistically significantly higher than in the COVID-19 negative group (81.1% vs. 70.7%, *p* = 0.009). The biggest difference was observed in those who took a daily dose of both iron and folic acid (52.6% vs. 33.9%). It was observed that newborns of mothers with anemia were also more likely to have anemia. In this case, the stratification by COVID-19 status determined a significant difference in the number of newborns identified with anemia (31.6% vs. 21.7%, *p* = 0.043). Another divergence between the two study groups was the newborn birth weight and APGAR scores. Therefore, the birth weight and APGAR score were statistically lower in the newborns of mothers with COVID-19 (*p* = 0.027), respectively (*p* = 0.029).

**Figure 1:**
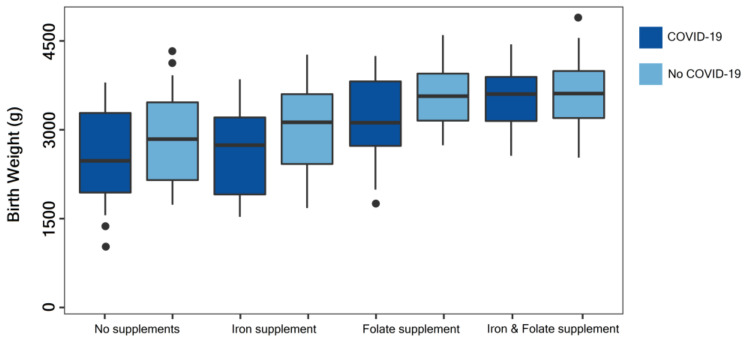
Boxplot comparison of birth weight by nutritional supplementation in newborns from COVID-19 positive vs. COVID-19 negative mothers. Data were stratified by nutritional supplements taken during pregnancy and evaluated using the Kruskal-Wallis test. Median values and interquartile range are represented inside the box; minimum, maximum, and outliers are shown outside the box as “●”.

By evaluating the newborns’ birth weight by COVID-19 and no COVID-19 groups, we observed the lowest median value in the group of newborns from mothers with COVID-19 who did not take iron or folate supplements during pregnancy (median birth weight = 2590 g). The highest median value of birth weight was observed in the group of newborns from mothers without a SARS-CoV-2 infection who followed a nutritional supplementation with iron and folate (median birth weight = 3340 g), as seen in Figure 1. There was a significant difference in data stratified by nutritional supplementation between groups (*p* = 0.022) but not within groups (*p* = 0.094).

The laboratory profile of pregnant women with anemia indicated that SARS-CoV-2 infection is associated with worsening anemia (Table 2). We observed statistically significant differences between groups of COVID-19 positive and COVID-19 negative patients, where the red blood cell count, hemoglobin, ferritin, sideremia, transferrin saturation, and reticulocyte count were significantly lower in COVID-19 positive pregnant women with anemia. The white blood cell count and haptoglobin levels were significantly elevated in the COVID-19 positive group.

Table 3 describes the comparison by the status of iron deficiency anemia between mothers infected with SARS-CoV-2 during pregnancy. It was observed that puerperal infections occurred in a statistically significantly higher proportion in mothers with anemia (52.5% vs. 27.3%, *p* = 0.015). A total of 42.6% of pregnancies from mothers with COVID-19 and anemia terminated with emergency c-section, in comparison with 18.2% in the other group (*p* = 0.009). Additionally, the analysis found a significant difference in fetal growth, since 35.0% were small for gestational age in the group of pregnancies with anemia and SARS-CoV-2 infection, compared with 14.5% in pregnancies with SARS-CoV-2 infection but no anemia (*p* = 0.019).

**Figure 2:**
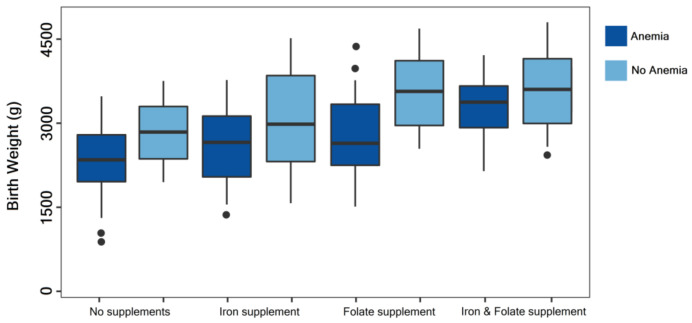
Boxplot comparison of birth weight in newborns from COVID-19 mothers with and without anemia. Data were stratified by nutritional supplements taken during pregnancy and evaluated using the Kruskal-Wallis test. Median values and interquartile range are represented inside the box; minimum, maximum, and outliers are shown outside the box as “●”.

It was observed that newborn birth weight varied significantly by the status of maternal anemia (*n* = 40) in mothers who suffered a SARS-CoV-2 infection during pregnancy (*n* = 95). We observed the lowest median value in the group of newborns from mothers with anemia who did not take iron or folate supplements during pregnancy (median birth weight = 2410 g). The highest median value of birth weight was observed in the group of newborns from mothers without anemia who followed a nutritional supplementation with iron and folate (median birth weight = 3480 g), as seen in Figure 2. There was a significant difference between data stratified by nutritional supplementation between groups (*p* < 0.001) and within groups (*p* = 0.006).

Lastly, the correlation analysis determined significant associations between maternal iron deficiency anemia and maternal and neonatal outcomes in both the COVID-19 positive and negative groups (Table 4). There was a significantly strong negative association between iron and folic acid supplementation with iron deficiency in COVID-19 positive pregnancies (*r* = −0.646, *p* = 0.005), and a lower negative association in pregnant women who followed an iron only nutritional supplementation (*r* = −0.310, *p* = 0.033). Anemia had a statistically significant positive correlation with severe SARS-CoV-2 infection, puerperal infection, transfusion necessity, emergency c-section, newborns small for gestational age. In the same manner, it was negatively correlated with birth weight and APGAR scores in COVID-19 positive mothers.

## 4. Discussion

Our study sets an important step forward in understanding the implications of nutritional supplementation for iron deficiency anemia in pregnant women who suffered a SARS-CoV-2 infection during their pregnancy. This retrospective research allowed for comprehensive data collection of blood samples and clinical outcomes in the target population over a long time period. The primary merit of this study is represented by a comparison of laboratory profiles of pregnant women by the trimester of COVID-19 diagnosis to determine how SARS-CoV-2 is involved in existent alterations. Thus, we found evidence that anemia was more likely to be present in patients infected with SARS-CoV-2, and the severity of disease was higher in the pregnancies with a diagnosis of iron deficiency anemia. These findings are consistent with existing literature describing COVID-19 as a significant factor associated with anemia [26]. Another important consequence worth mentioning was the significantly lower birth weight of newborns from mothers with COVID-19 and anemia, even after adjusting for confounders.

### 4.1. Review of the Literature

Citing the existing literature, it is common that anemia in pregnancy is related to iron deficiency and there will always be increased strain on iron reserves as pregnancy continues. Pairing the complete blood count with serum ferritin minimizes the danger of iron supplementation in patients with probable iron excess indicated by high serum ferritin. Although tolerance and compliance are possible difficulties, employing a modest dosage is perhaps better tolerated. Starting early enhances the likelihood of having adequate storage later in pregnancy and seeks to limit the requirement for intravenous iron [27].

Anemia is also relatively prevalent comorbidity in patients with SARS-CoV-2 infection, occurring in about 25% of infected individuals, as a meta-analysis discovered [28]. More critically, individuals infected with SARS-CoV-2 who were anemic had a roughly 70% increased risk of mortality in the near term compared to those who were not. Additionally, COVID-19 patients with hemoglobin higher than 10.0 g/dL had a decreased risk of death than those with hemoglobin levels lower than 10/0 g/dL [29]. From a pathophysiological standpoint, hemoglobin concentration is a critical indicator of the blood’s oxygen-carrying ability. As a result, anemia may further impair oxygen transport to peripheral tissue in COVID-19 patients with increased oxygen demand owing to pneumonia [30,31].

In light of our findings, it is mandatory to cite the existing guidelines referring to the management of iron deficiency anemia during pregnancy, hoping that fewer complications will occur during the present pandemic and the future. Therefore, the Centers for Disease Control and Prevention and the World Health Organization recommend screening asymptomatic pregnant women for iron deficiency anemia using serum hemoglobin and ferritin levels, as well as universal iron supplementation with 30–60 mg/day of elemental iron during pregnancy [32,33]. They recommend increasing the amount of elemental iron to 60–120 mg/day in pregnancies associated with anemia [34]. Despite iron pills being the most common type of supplementation, recent studies debate the use of lactoferrin in treating anemia in pregnant women [35], with potential benefits in the context of preventing and managing COVID-19 [36]. The Network for the Advancement of Patient Blood Management, Hemostasis, and Thrombosis consensus statement also recommends daily oral iron supplementation with 30–60 mg/day in areas where there is a high prevalence of anemia during pregnancy and screening for iron deficiency in all pregnancies at risk of iron deficiency [37]. Additionally, the same consensus recommends intravenous iron in pregnancies associated with iron deficiency anemia and hemoglobin levels lower than 8.0 g/dL, or newly diagnosed iron deficiency anemia after 34 weeks of gestation [37]. Although by UK guidelines, intravenous iron is recommended in pregnancy if a woman is diagnosed with iron insufficiency after 34 weeks of gestation and has a hemoglobin level of 10.0 g/dL or if she is intolerant to or does not respond to oral iron. In contrast, intravenous iron administration was not considered for our patients, nor is it a common approach in our country. Moreover, the United States Preventative Task Force notes a scarcity of data to justify widespread prenatal screening or iron supplementation, perhaps as a consequence of this uncertainty, fewer than a third of American obstetrician-gynecologists routinely supplement pregnant women with iron, which is similar to the situation that we are facing in Romania [38,39].

Although the necessity for blood transfusions was very low in our studied cohort, there was a significant increase in the group of patients with COVID-19 and anemia diagnosed during pregnancy. It is known that untreated anemia during pregnancy or at the time of birth increases the likelihood of having blood transfusions due to additional demands, such as blood loss [40]. Iron is administered intravenously, and blood is transfused in a hospital environment. This should be avoided in conditions caused by the COVID-19 pandemic, since it entails treatment in a hospital environment. A more proactive strategy is essential in light of the present pandemic situation. In view of the ongoing SARS-CoV-2 pandemic, one study suggests that all women with hemoglobin less than 12.0 g/dL should have low-dose iron supplementation, even if their serum ferritin is normal [8]. The same study recommends that iron should also be supplied if the serum ferritin level is less than 30 mcg/L, regardless of the hemoglobin level. This suggests that around 10% of pregnant women will be prescribed iron treatment solely based on their hemoglobin level at the time of evaluation.

### 4.2. Strengths and Limitations

One limitation is the statistical power in comparing pregnant women with SARS-CoV-2 infection by their status of anemia, since only 95 patients were included in the statistical analysis, and the incidence of complications was generally low. Furthermore, the study groups were not case-matched. Another limitation of the study can be the significant difference in nutritional supplementation that was found between groups of pregnant women with and without COVID-19. Here, we can consider the SARS-CoV-2 as a confounding factor that might influence the decision to take more supplements, hoping to recover faster and avoid unknown disease complications.

## 5. Conclusions

This study proved the importance of careful management and nutritional supplementation for iron deficiency anemia in pregnant women during the COVID-19 pandemic since the additive effect of SARS-CoV-2 infection on the impaired maternal health caused by anemia significantly increases the risks for negative outcomes for the mother and the newborn.

## Figures and Tables

**Table 1:** General characteristics of pregnancies associated with SARS-CoV-2.

Variables *	COVID-19 Positive (*n* = 95)	COVID-19 Negative (*n* = 351)	*p*
**Maternal characteristics**			
**Age**			0.269
<25	8 (8.4%)	44 (12.5%)	
25–34	65 (68.4%)	247 (70.4%)	
>34	22 (23.2%)	60 (17.1%)	
**Gravidity**			0.826
1	49 (51.6%)	171 (48.7%)	
2	29 (30.5%)	108 (30.8%)	
≥3	17 (17.9%)	72 (20.5%)	
**Parity**			0.296
1	56 (58.9%)	189 (53.8%)	
2	32 (33.7%)	116 (33.0%)	
≥3	7 (7.4%)	46 (13.1%)	
**Complications**			
Anemia	40 (42.1%)	103 (29.3%)	0.018
Gestational hypertension	4 (4.2%)	12 (3.4%)	0.712
Gestational diabetes mellitus	5 (5.3%)	20 (5.7%)	0.870
PROM	10 (10.5%)	22 (6.3%)	0.153
**Nutritional supplementation**			0.009
No Iron/Folic Acid	18 (18.9%)	92 (26.2%)	
Iron	17 (17.9%)	98 (27.9%)	
Folic Acid	10 (10.6%)	42 (12.0%)	
Iron and Folic Acid	50 (52.6%)	119 (33.9%)	
**Neonatal characteristics**			
**Complications**			
Anemia	30 (31.6%)	76 (21.7%)	0.043
Puerperal infection	7 (7.4%)	18 (5.3%)	0.399
Premature birth	14 (14.7%)	28 (7.9%)	0.045
Malformations	2 (2.1%)	6 (1.7%)	0.796
**Birth weight**			0.027
<1500 g	4 (4.2%)	5 (1.4%)	
1500–2500 g	15 (15.8%)	31 (8.8%)	
>2500 g	76 (80.0%)	315 (89.7%)	
**APGAR score**			0.029
≥9	75 (78.8%)	308 (87.7%)	
7–8	10 (10.6%)	29 (8.3%)	
≤6	10 (10.6%)	14 (4.0%)	

* Data presented as *n* (frequency) unless specified differently.

**Table 2:** Laboratory profile of pregnant women with iron deficiency anemia by the history of SARS-CoV-2 infection.

Laboratory Data *	Normal Range	COVID-19 Positive (*n* = 40)	COVID-19 Negative (*n* = 103)	*p*
RBC (millions/mm^3^)	4.0–5.0	3.4 ± 1.6	3.8 ± 1.2	0.012
Platelets (thousands/mm^3^)	150–450	168 ± 31	171 ± 36	0.643
WBC (thousands/mm^3^)	4.0–10.0	12.6 ± 4.8	7.5 ± 2.2	<0.001
Hemoglobin (g/dL)	11.5–14.0	10.1 ± 2.9	11.0 ± 2.1	0.041
Hematocrit (g/dL)	35–44	33.4 ± 5.3	34.7 ± 4.6	0.148
Mean Corpuscular Volume (fL)	80–96	88.7 ± 9.5	87.2 ± 9.0	0.379
Ferritin (ng/mL)	30–150	21.4 ± 4.2	23.3 ± 4.6	0.024
Sideremia (µg/dL)	50–170	42.8 ± 6.2	45.3 ± 5.9	0.026
Transferrin (saturation %)	15–45	12.3 ± 2.5	13.8 ± 2.0	<0.001
Fe (μmol/L)	10–30	7.6 ± 2.1	8.8 ± 2.3	0.004
Reticulocyte count (%)	0.5–2.5	0.6 ± 0.2	0.7 ± 0.2	0.008
Serum folate (nmol/L)	10–45	15.7 ± 3.1	16.2 ± 3.0	0.376
Total iron-binding capacity (µg/dL)	41–73	68.0 ± 9.1	65.4 ± 8.6	0.112
Haptoglobin (g/L)	0.3–2.0	3.3 ± 0.4	0.5 ± 0.1	<0.001

* Data presented as mean ± SD, unless specified differently; RBC—Red Blood Cells; WBC—White Blood Cells.

**Table 3:** Maternal and neonatal outcomes associated with SARS-CoV-2 infection stratified by presence of anemia.

Variables *	Total (*n* = 95)	Anemia (*n* = 40)	No Anemia (*n* = 55)	*p*
**Maternal outcomes**				
Severe COVID-19	5 (5.3%)	3 (7.5%)	2 (3.6%)	0.405
Puerperal infection	7 (26.3%)	6 (15.0%)	1 (1.8%)	0.015
Postpartum hemorrhage	18 (18.9%)	10 (25.0%)	8 (14.5%)	0.199
Antepartum hemorrhage	18 (18.9%)	9 (22.5%)	9 (16.4%)	0.451
Transfusion necessity	5 (5.3%)	4 (10.0%)	1 (1.8%)	0.077
Abnormal placentation	9 (9.5%)	4 (10.0%)	5 (9.1%)	0.881
PROM **	10 (10.5%)	7 (17.5%)	3 (5.4%)	0.058
Gestational hypertension	4 (4.2%)	3 (7.5%)	1 (1.8%)	0.173
Gestational diabetes mellitus	5 (5.3%)	4 (10.0%)	1 (1.8%)	0.077
Pre-eclampsia	4 (4.2%)	2 (5.0%)	2 (3.6%)	0.743
Emergency c-section	27 (28.4%)	17 (42.6%)	10 (18.2%)	0.009
**Neonatal outcomes**				
Small for gestational age	22 (23.2%)	14 (35.0%)	8 (14.5%)	0.019
Low birth weight	19 (20.0%)	11 (27.5%)	8 (14.5%)	0.119
Prematurity	14 (14.7%)	9 (22.5%)	5 (9.1%)	0.068
Sepsis	5 (5.3%)	3 (7.5%)	2 (3.6%)	0.405
Low APGAR score (<7)	10 (10.5%)	6 (15.0%)	4 (7.3%)	0.225

* Data presented as *n* (frequency) unless specified differently; ** Premature Rupture of Membranes.

**Table 4:** Correlation of iron deficiency anemia with nutritional supplementation and pregnancy outcomes by COVID-19 status.

Variables *	COVID-19 Positive		COVID-19 Negative	
	*****r*****	***p***	***r***	***p***
**Nutritional supplementation**				
No Iron/Folic Acid	0.229	0.148	0.204	0.319
Iron	−0.310	0.033	−0.248	0.127
Folic Acid	−0.243	0.106	−0.255	0.202
Iron and Folic Acid	−0.646	0.005	−0.410	0.024
**Maternal outcomes**				
Severe COVID−19	0.332	0.044	−	−
Puerperal infection	0.508	0.002	0.426	0.017
Postpartum hemorrhage	0.204	0.313	0.148	0.352
Antepartum hemorrhage	0.197	0.390	0.124	0.479
Transfusion necessity	0.691	0.001	0.662	0.001
Abnormal placentation	0.301	0.446	0.347	0.320
PROM **	0.374	0.098	0.355	0.127
Gestational hypertension	0.126	0.670	0.134	0.558
Gestational diabetes mellitus	0.108	0.642	0.128	0.643
Pre-eclampsia	0.214	0.573	0.246	0.540
Emergency c−section	0.497	0.038	0.366	0.192
**Neonatal outcomes**				
Small for gestational age	0.418	0.045	0.338	0.173
Birth weight	−0.525	0.001	−0.386	0.010
Prematurity	0.429	0.040	0.347	0.095
Sepsis	0.321	0.127	0.304	0.144
APGAR score	−0.431	0.008	−0.315	0.042

* Data reported as Spearman’s correlation coefficient (*r*) and probability (*p*-value); ** Premature Rupture of Membranes.

## Data Availability

The data presented in this study are available on request from the corresponding author.
